# Altered Brain Arginine Metabolism and Polyamine System in a P301S Tauopathy Mouse Model: A Time-Course Study

**DOI:** 10.3390/ijms23116039

**Published:** 2022-05-27

**Authors:** Hannah Mein, Yu Jing, Faraz Ahmad, Hu Zhang, Ping Liu

**Affiliations:** 1 Brain Health Research Centre, Department of Anatomy, School of Biomedical Sciences, University of Otago, Dunedin P.O. Box 56, New Zealand; hannah.mein@otago.ac.nz (H.M.); rena.jing@otago.ac.nz (Y.J.); far.ahmad14@gmail.com (F.A.); 2 Brain Health Research Centre, School of Pharmacy, University of Otago, Dunedin P.O. Box 56, New Zealand; hu.zhang@otago.ac.nz

**Keywords:** tauopathy, PS19 mice, tau, arginine, polyamine, spermine, polyamine stress response, hippocampus

## Abstract

Altered arginine metabolism (including the polyamine system) has recently been implicated in the pathogenesis of tauopathies, characterised by hyperphosphorylated and aggregated microtubule-associated protein tau (MAPT) accumulation in the brain. The present study, for the first time, systematically determined the time-course of arginine metabolism changes in the *MAPT* P301S (PS19) mouse brain at 2, 4, 6, 8 and 12 months of age. The polyamines putrescine, spermidine and spermine are critically involved in microtubule assembly and stabilization. This study, therefore, further investigated how polyamine biosynthetic and catabolic enzymes changed in PS19 mice. There were general age-dependent increases of L-arginine, L-ornithine, putrescine and spermidine in the PS19 brain (particularly in the hippocampus and parahippocampal region). While this profile change clearly indicates a shift of arginine metabolism to favor polyamine production (a polyamine stress response), spermine levels were decreased or unchanged due to the upregulation of polyamine retro-conversion pathways. Our results further implicate altered arginine metabolism (particularly the polyamine system) in the pathogenesis of tauopathies. Given the role of the polyamines in microtubule assembly and stabilization, future research is required to understand the functional significance of the polyamine stress response and explore the preventive and/or therapeutic opportunities for tauopathies by targeting the polyamine system.

## 1. Introduction

The microtubule-associated protein tau (MAPT or “tau”) is a key regulator of microtubule dynamics and is critically involved in axonal elongation and transport processes [1,2,3]. The intracellular accumulation of hyperphosphorylated and aggregated tau is a prominent histopathological feature for a group of neurodegenerative disorders termed tauopathies, including Alzheimer’s disease (AD) and frontotemporal dementia (FTD) [1,4,5]. The link between *MAPT* mutations and FTD indicates a specific contribution of tau abnormalities to frontotemporal lobar degeneration [6]. Transgenic mouse lines bearing *MAPT* mutations have, therefore, been used to mimic human frontotemporal lobar degeneration [7,8]. PS19 mice expressing the *MAPT* P301S mutation, for example, progressively develop pathogenic tau species, neuronal loss and cognitive impairments [7,9,10,11,12,13].

**Figure 1:**
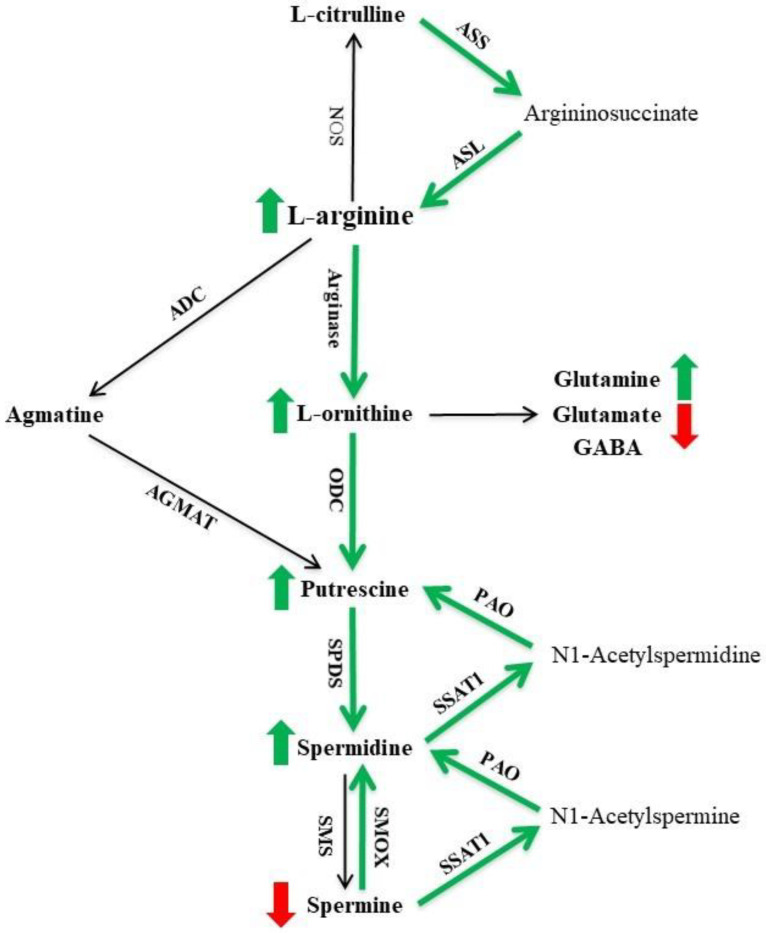
L-arginine metabolic pathways and the polyamine system. L-arginine is metabolized by nitric oxide synthase (NOS), arginase and arginine decarboxylase (ADC) to form several bioactive molecules and can be de novo synthesized by argininosuccinate synthetase (ASS) and argininosuccinate lyase (ASL) from L-citrulline (see text for detailed description). Overall, we found genotype- and age-related increases in L-arginine, L-ornithine, glutamine, putrescine and spermidine (indicated by short green arrows) and decreases in glutamate and spermine (indicated by short red arrows) levels in the brain of PS19 mice, indicating a shift of L-arginine metabolism towards the arginase and polyamine system. Moreover, PS19 mice displayed increased levels of mRNA and/or protein expression of ASS, ASL, arginase I and II, ornithine decarboxylase (ODC), spermidine synthase (SPDS), spermine oxidase (SMOX), spermidine/spermine-N^1^-acetyltransferase-1 (SSAT1) and polyamine oxidase (PAO), suggesting upregulated citrulline recycling and polyamine retro-conversion (indicated by long green arrows). AGMAT, agmatinase; SMS, spermine synthase. Adapted from [28].

L-arginine is a metabolically versatile semi-essential amino acid. Its de novo synthesis involves the conversion of L-citrulline by argininosuccinate synthetase (ASS) and argininosuccinate lyase (ASL), the so-called L-citrulline recycling pathway (Figure 1) [14,15,16]. In the brain, L-arginine can be metabolized by nitric oxide synthase (NOS) to produce L-citrulline and nitric oxide (NO), by arginase to form L-ornithine and urea, and by arginine decarboxylase (ADC) to generate agmatine (Figure 1). L-ornithine can be further metabolized to form the polyamine putrescine by ornithine decarboxylase (ODC) and to produce glutamate, γ-aminobutyric acid (GABA) and glutamine via an alternative pathway (Figure 1) [14,15,16]. The polyamines putrescine, spermidine and spermine play important roles in cell proliferation and differentiation, synthesis of DNA, RNA and proteins, protein phosphorylation, signal transduction and the regulation of neurotransmitter receptors [17,18,19,20,21]. Recent research has shown that polyamines are critically involved in microtubule assembly and stabilization [22,23,24], and the higher-order polyamines (spermine in particular) protect against tau fibrilization and reduce the formation of toxic tau species [25,26]. As illustrated in Figure 1, several enzymes are involved in the formation of polyamines. The de novo synthesis pathway includes ODC (the rate-limiting enzyme to form putrescine), spermidine synthase (SPDS, to generate spermidine from putrescine) and spermine synthase (SMS, to generate spermine from spermidine), whereas the retro-conversion pathways involve spermine oxidase (SMOX) to convert spermine to spermidine, and spermidine/spermine-N^1^-acetyltransferase-1 and polyamine oxidase (SSAT1 and PAO, respectively) to produce spermidine or putrescine with the formation of acetylated polyamine intermediaries [17,18]. Moreover, agmatine (decarboxylated arginine) can be metabolised to form putrescine by agmatinase (AGMAT; Figure 1), and itself can also influence ODC activity and regulate polyamine transport [15,17,27].

Under physiological situations, the NOS pathway is the predominate L-arginine metabolic pathway, as NOS has an approximately 1000-fold greater affinity for L-arginine than arginase and the endogenous agmatine level in the brain is very low (only about 0.2 to 0.4 μg/g) [14]. In AD brains, interestingly, arginine metabolism is altered with a shift towards the arginase pathway along with various changes in polyamine levels and upregulated gene expression of the polyamine retro-conversion enzymes [29,30,31]. Sandusky-Beltran et al. [26] reported that rTg4510 mice expressing the *MAPT* P301L mutation displayed increased putrescine and acetylspermidine levels in the whole brain tissue at 8 months of age, and reduced ODC and SMS expression, but increased SPDS, SSAT1 and SMOX expression in the hippocampus at 12 months of age, indicating the disrupted polyamine homeostasis in mice with a tau mutation. Using PS19 mice bearing the *MAPT* P301S mutation, we previously investigated how the arginine metabolic profile changed in the brain at 4, 8 and 12–14 months of age [28]. Intriguingly, there were significantly increased levels of L-ornithine (in all three age groups) and the polyamines putrescine and spermidine (particularly in older age groups), but a trend of reduced spermine levels in the hippocampus of PS19 mice. Again, these findings demonstrate a clear shift of arginine metabolism to favour the arginase-polyamine system in PS19 tau mice, perhaps aiming to produce more polyamines (spermine in particular) to combat tau pathology. However, the system failed to produce more spermine in the hippocampus of PS19 mice even at 12–14 months of age, when high levels of putrescine and spermidine were present. We postulate that such a failure might be due to impaired spermine biosynthesis and/or enhanced spermine retro-conversion to spermidine.

It has been shown that the de novo synthesis pathway is the safest way to produce polyamines, as the indirect retro-conversion pathway can generate toxic by-products [17,32,33]. Given the role of polyamines in microtubule assembly and stabilization [22,23,24], the existing evidence implicates altered polyamine homeostasis in the pathogenesis of tauopathies. The present time-course study was designed to systematically investigate how brain arginine metabolism, particularly the polyamine system (including both the de novo synthesis and retro-conversion pathways), changed in PS19 mice during their progression from prodromal to severe disease-like stages, using mice at 2 (mild behavioral deficits), 4 (synaptic loss), 6 (tau aggregates in the brain), 8 (hippocampal neuronal loss) and 12 (widespread atrophy) months of age [7,12]. In order to analyze correlations between the brain L-arginine metabolite changes in the PS19 mice and their behavioral deficits, we used a cohort of animals with confirmed age-related accumulation of phosphorylated tau (*p*-tau) species and behavioral impairments [12].

## 2. Results

### 2.1. L-Arginine and Its Downstream Metabolites

We utilized the high-performance liquid chromatographic (HPLC) and liquid chromatography/mass spectrometric (LC/MS) assays to measure the concentrations of L-arginine and nine of its downstream metabolites (L-citrulline, L-ornithine, glutamine, glutamate, GABA, agmatine, putrescine, spermidine and spermine) in the frontal cortex (FC), hippocampus (HP), parahippocampal region (PH), striatum (ST) and cerebellum (CE) of PS19 mice and litter-matched wildtype (WT) mice at 2, 4, 6, 8 and 12 months of age. Moreover, the glutamine/glutamate and spermidine/spermine ratios were also calculated for all five brain regions, as these ratios reflect changes in the glutamate–glutamine cycle and cellular function, respectively [34,35,36,37].

#### 2.1.1. L-Arginine

**Figure 2:**
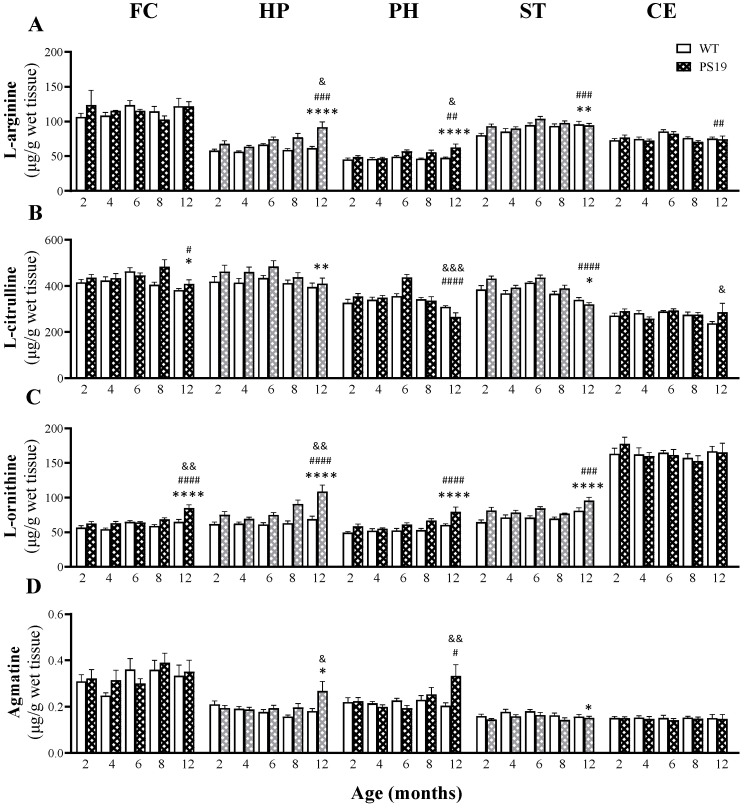
Mean (± SEM) levels of L-arginine (**A**) and its three direct metabolites L-citrulline (**B**), L-ornithine (**C**) and agmatine (**D**) in the frontal cortex (FC), hippocampus (HP), parahippocampal region (PH), striatum (ST) and cerebellum (CE) of wildtype (WT) and PS19 mice at 2, 4, 6, 8 and 12 months of age (*n* = 7–9/genotype/age). ^&^ indicates a significant genotype and age interaction at ^&^ *p* < 0.05, ^&&^ *p* < 0.01 or ^&&&^ *p* < 0.001. ^#^ indicates a significant age effect at ^#^ *p* < 0.05, ^##^ *p* < 0.01, ^###^ *p* < 0.001 or ^####^ *p* < 0.0001. * indicates a significant genotype effect at * *p* < 0.05, ** *p* < 0.01 or **** *p* < 0.0001.

Figure 2A illustrates the levels of L-arginine in five brain regions of WT and PS19 mice at five age points. For the frontal cortex, there were no significant effects of genotype, age and their interaction (all F ≤ 1). In terms of the hippocampus and parahippocampal region, we observed significant effects of genotype (HP: F(1,65) = 38.27, *p* < 0.0001; PH: F(1,67) = 23.90, *p* < 0.0001), age (HP: F(4,65) = 5.90 *p* = 0.0004; PH: F(4,67) = 5.12, *p* = 0.0012) and their interaction (HP: F(4,65) = 3.18, *p* = 0.019; PH: F(4,67) = 2.68, *p* = 0.039), with increased levels of L-arginine with age particularly in PS19 mice. Moreover, L-arginine was increased 31–49% in the hippocampus of 8–12 PS19 mice and 15–31% in the parahippocampal region of 6–12-months-old PS19 mice, when compared to their age-matched WT littermates. Regarding the striatum, we found significant effects of genotype (F(1,66) = 8.02, *p* = 0.0061) and age (F(4,66) = 5.73, *p* = 0.0005), but not their interaction (F < 1.5), with 9% and 17% increases in 2- and 6-month-old PS19 mice, respectively. For the cerebellum, there was a significant effect of age (F(4,67) = 5.13, *p* = 0.0011), but not genotype or interaction (both F < 1), with increased levels of L-arginine with age (peak at 6 months) regardless of genotype.

#### 2.1.2. L-Citrulline

The levels of L-citrulline in five brain regions of WT and PS19 mice at five age points are presented in Figure 2B. For the frontal cortex, there were significant effects of genotype (F(1,66) = 4.49, *p* = 0.038) and age (FC: F(4,66) = 3.06, *p* = 0.022), but not their interaction (F = 2), with a higher level in 8-month-old PS19 mice (19%) relative to their age-matched WT littermates. Regarding the hippocampus, we found a significant effect of genotype (F(1,68) = 7.32, *p* = 0.0086), but not age or their interaction (both F < 2), with a general pattern of small increases (≤ 11%) in 2–8-months-old PS19 mice. In terms of the parahippocampal region, there were significant effects of age (F(4,66) = 17.75, *p* < 0.0001) and its interaction with genotype (F(4,66) = 6.37, *p* = 0.0002), but not genotype (F(1,66) = 2.78, *p* = 0.10), with a 22% increase but a 14% reduction in 6- and 12-month-old PS19 mice, respectively. For the striatum, we observed significant effects of genotype (F(1,66) = 6.52, *p* = 0.013) and age (F(4,66) = 16.62, *p* < 0.0001), but not their interaction (F < 2), with age-related reductions irrespective of genotype and a 12% increase in 2-month-old PS19 mice relative to their age-matched WT littermates. Regarding the cerebellum, there was a significant genotype and age interaction (F(4,67) = 3.56, *p* = 0.011), but no effect of genotype (F(1,67) = 2.45, *p* = 0.12) or age (F(4,67) = 2.34, *p* = 0.064), with a 20% increase in 12-month-old PS19 mice.

#### 2.1.3. L-Ornithine

Figure 2C illustrates the levels of L-ornithine in five brain regions of WT and PS19 mice at five age points. In general, we observed more sustained changes across the brain regions, except for the cerebellum, with no significant effects of genotype, age and their interaction (all F < 1). For both the frontal cortex and hippocampus, there were significant effects of genotype (FC: F(1,66) = 24.87, *p* < 0.0001; HP: F(1,67) = 51.94, *p* < 0.0001), age (FC: F(4,66) = 10.57, *p* < 0.0001; HP: F(4,67) = 8.33, *p* < 0.0001) and their interaction (FC: F(4,66) = 3.63, *p* = 0.0098; HP: F(4,67) = 4.21, *p* = 0.0042). PS19 mice displayed increased levels of L-ornithine in their frontal cortex (16–30%) at the age points of 4, 8 and 12 months and in the hippocampus at 2 (21%), 6 (22%), 8 (44%) and 12 (58%) months of age, in relation to their age-matched WT littermates. Regarding the parahippocampal region and striatum, we found significant effects of genotype (PH: F(1,67) = 29.04, *p* < 0.0001; ST: F(1,67) = 29.70, *p* < 0.0001) and age (PH: F(4,67) = 8.58, *p* < 0.0001; ST: F(4,67) = 6.73, *p* < 0.0001), but not their interaction (both F < 2), with higher levels in the parahippocampal region of PS19 mice at 2 (18%), 8 (26%) and 12 (32%) months of age and 10–26% increases in the striatum in 2–12-months-old PS19 mice.

#### 2.1.4. Agmatine

The levels of agmatine in five brain regions of WT and PS19 mice at five age points are presented in Figure 2D. For both the frontal cortex and cerebellum, we found no significant effects of genotype, age and their interaction (all F ≤ 2). In terms of the hippocampus, there were significant effects of genotype (F(1,67) = 5.36, *p* = 0.024) and its interaction with age (F(4,67) = 2.87, *p* = 0.03), but not age (F(4,67) = 2.36, *p* = 0.06), with increased levels in 8 (25%)- and 12 (47%)-month-old PS19 mice relative to their WT littermates. Regarding the parahippocampal region, we observed significant effects of age (F(4,66) = 2.80, *p* = 0.033) and interaction (F(4,66) = 4.24, *p* = 0.0041), but not genotype (F(1,66) = 2.67, *p* = 0.11), with a 63% increase in 12-month-old PS19 mice. For the striatum, there was a significant effect of genotype (F(1,63) = 6.38, *p* = 0.014), but not age or their interaction (both F ≤ 2), with a small (4–12%) reduction in PS19 mice at all five age points.

#### 2.1.5. Glutamine

**Figure 3:**
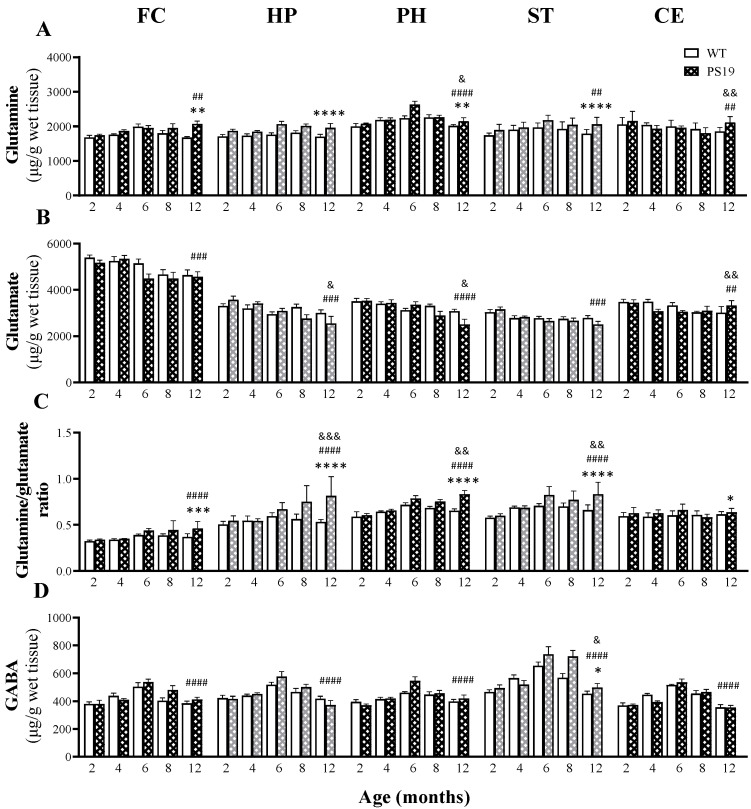
Mean (± SEM) levels of glutamine (**A**), glutamate (**B**), the glutamine/glutamate ratio (**C**) and GABA (**D**) in the frontal cortex (FC), hippocampus (HP), parahippocampal region (PH), striatum (ST) and cerebellum (CE) of wildtype (WT) and PS19 mice at 2, 4, 6, 8 and 12 months of age (*n* = 7–9/genotype/age). ^&^ indicates a significant genotype and age interaction at ^&^ *p* < 0.05, ^&&^ *p* < 0.01 or ^&&&^ *p* < 0.001. ^#^ indicates a significant age effect at ^##^ *p* < 0.01, ^###^ *p* < 0.001 or ^####^ *p* < 0.0001. * indicates a significant genotype effect at * *p* < 0.05, ** *p* < 0.01, *** *p* < 0.001 or **** *p* < 0.0001.

Figure 3A illustrates the levels of glutamine in five brain regions of WT and PS19 mice at five age points. For both the frontal cortex and striatum, we found significant effects of genotype (FC: F(1,67) = 9.37, *p* = 0.0032; ST: F(1,67) = 19.26, *p* < 0.0001) and age (FC: F(4,67) = 3.98, *p* = 0.0059; ST: F(4,67) = 5.02, *p* = 0.001), but not their interaction (FC: F(4,67) = 2.45, *p* = 0.055; ST: F < 1), with increased levels in the frontal cortex (24%) in 12-month-old PS19 mice and in the striatum (11–15%) in 6- and 12-month-old PS19 mice. Moreover, we observed increased glutamine levels with age (peak at 6 months) in the striatum irrespective of genotype. Regarding the hippocampus, there was a significant effect of genotype (F(1,67) = 24.92, *p* < 0.0001), but not age or their interaction (both F < 2), with 11–17% increases in 6–12-months-old PS19 mice. For the parahippocampal region, we found significant effects of genotype (F(1,65) = 7.57, *p* = 0.0077), age (F(4,65) = 10.00, *p* < 0.0001) and their interaction (F(4,65) = 2.80, *p* = 0.033), with age-related increases (peak at 6 months) and a 17% increase in 6-month-old PS19 mice relative to their age-matched WT littermates. In terms of the cerebellum, there were significant effects of age (F(4,67) = 4.58, *p* = 0.0025) and its interaction with genotype (F(4,67) = 3.81, *p* = 0.0075), but not genotype (F < 1), with age-related decreases in both genotypes, except for a 14% increase in 12-month-old PS19 mice.

#### 2.1.6. Glutamate

The levels of glutamate in five brain regions of WT and PS19 mice at five age points are presented in Figure 3B. Regarding the frontal cortex and striatum, there was a significant effect of age (FC: F(4,67) = 6.84, *p* = 0.0001; ST: F(4,67) = 6.41, *p* = 0.0002), but not genotype (FC: F(1,67) = 2.91, *p* = 0.093; ST: F ≤ 1) or their interaction (both F ≤ 1), with reduced levels with age regardless of the genotype. In terms of the hippocampus, parahippocampal region and cerebellum, we found significant effects of age (HP: F(4,67) = 5.96, *p* = 0.0004; PH: F(4,67) = 9.28, *p* < 0.0001; CE: F(4,66) = 4.85, *p* = 0.0017) and its interaction with genotype (HP: F(4,67) = 3.17, *p* = 0.019; PH: F(4,67) = 3.41, *p* = 0.013; CE: F(4,66) = 4.31, *p* = 0.0037), but not genotype (HP and CE F ≤ 1; PH: F(4,67) = 3.00, *p* = 0.09). Age-related reductions were evident in these regions in both genotypes, with more prominent changes in PS19 mice in the hippocampus and parahippocampal region at 8 and 12 months of age and in the cerebellum at the age points of 4 and 6 months.

#### 2.1.7. Glutamine/Glutamate Ratio

Figure 3C illustrates the glutamine/glutamate ratios in five brain regions of WT and PS19 mice at five age points. Regarding the frontal cortex, we observed significant effects of genotype (F(1,68) = 15.78, *p* = 0.0002) and age (F(4,68) = 11.24, *p* < 0.0001), but not their interaction (F < 2), with higher levels (13–25%) in PS19 mice at 6, 8 and 12 months of age in relation to their age-matched WT littermates. For the hippocampus, parahippocampal region and striatum, there were significant effects of genotype (HP: F(1,67) = 29.35, *p* < 0.0001; PH: F(1,67) = 24.13, *p* < 0.0001; ST: F(1,66) = 22.64, *p* < 0.0001), age (HP: F(4,67) = 7.90, *p* < 0.0001; PH: F(4,67) = 18.20, *p* < 0.0001; ST: F(4,66) = 16.11, *p* < 0.0001) and their interaction (HP: F(4,67) = 5.61, *p* = 0.0006; PH: F(4,67) = 4.23, *p* = 0.004; ST: F(4,66) = 3.85, *p* = 0.007). PS19 mice had higher ratios in the hippocampus at 6–12 months (13–53%), parahippocampal region at 12 months (27%), and striatum at 6–12 months (10–26%) relative to their age-matched WT controls. For the cerebellum, we found a significant genotype effect (F(1,68) = 6.17, *p* = 0.016), but not age or interaction (both F < 2), with a 10% increase in 6-month-old PS19 mice.

#### 2.1.8. GABA

The levels of GABA in five brain regions of WT and PS19 mice at five age points are presented in Figure 3D. For the frontal cortex, hippocampus, parahippocampal region and cerebellum, there were significant effects of age (FC: F(4,67) = 12.62, *p* < 0.0001; HP: F(4,67) = 13.64, *p* < 0.0001; PH: F(4,66) = 12.13, *p* < 0.0001; CE: F(4,65) = 29.85, *p* < 0.0001), but not genotype or their interaction (all F ≤ 3), with age-related increases in both genotypes (peak level at 6 months). In the striatum, we found significant effects of genotype (F(1,66) = 6.30, *p* = 0.015), age (F(4,66) = 17.98, *p* < 0.0001) and their interaction (F(4,67) = 2.53, *p* = 0.048), with age-related increases in both genotypes (peak at 6 months) and a 27% increase in 8-month-old PS19 mice relative to their WT littermates.

#### 2.1.9. Putrescine

**Figure 4:**
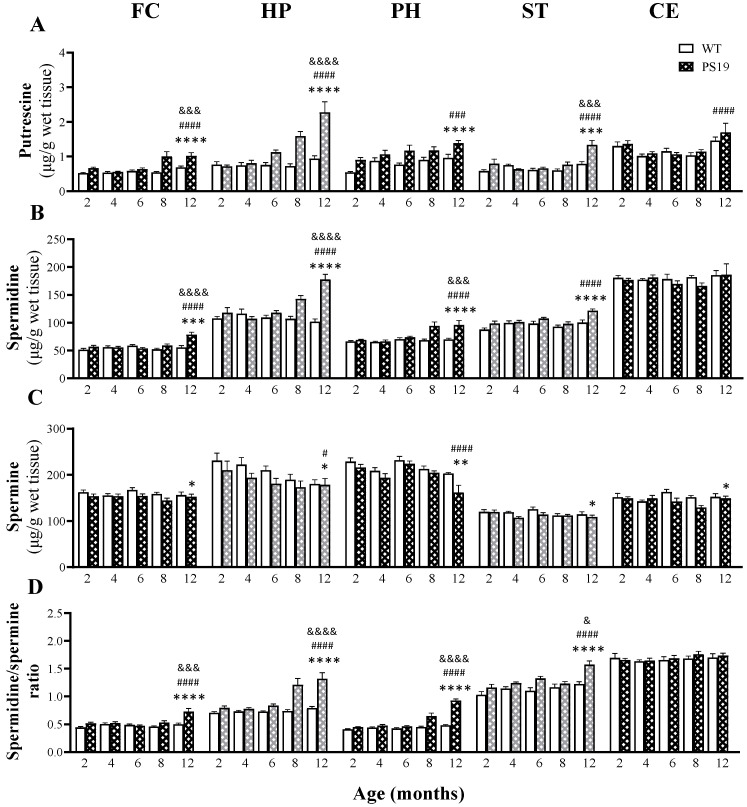
Mean (± SEM) levels of putrescine (**A**), spermidine (**B**), spermine (**C**) and the spermidine/spermine ratio (**D**) in the frontal cortex (FC), hippocampus (HP), parahippocampal region (PH), striatum (ST) and cerebellum (CE) of wildtype (WT) and PS19 mice at 2, 4, 6, 8 and 12 months of age (*n* = 7–9/genotype/age). ^&^ indicates a significant genotype and age interaction at ^&^ *p* < 0.05, ^&&&^ *p* < 0.001 or ^&&&&^ *p* < 0.0001. ^#^ indicates a significant age effect at ^#^ *p* < 0.05, ^###^ *p* < 0.001 or ^####^*p* < 0.0001. * indicates a significant genotype effect at * *p* < 0.05, ** *p* < 0.01, *** *p* < 0.001 or **** *p* < 0.0001.

Figure 4A illustrates the levels of putrescine in five brain regions of WT and PS19 mice at five age points. Regarding the frontal cortex, hippocampus and striatum, we found significant effects of genotype (FC: F(1,67) = 30.29, *p* < 0.0001; HP: F(1,67) = 49.12, *p* < 0.0001; ST: F(1,67) = 15.92, *p* = 0.0002), age (FC: F(4,67) = 10.05, *p* < 0.0001; HP: F(4,67) = 18.07, *p* < 0.0001; ST: F(4,67) = 12.41, *p* < 0.0001) and their interaction (FC: F(4,67) = 5.42, *p* = 0.0008; HP: F(4,67) = 12.10, *p* < 0.0001; ST: F(4,67) = 6.29, *p* = 0.0002). PS19 mice had increased levels of putrescine in the frontal cortex at 8 and 12 months (85% and 49% respectively), hippocampus at 6, 8 and 12 months (48%, 120% and 141%, respectively) and striatum at 2 and 12 months (37% and 70%, respectively). For the parahippocampal region, there were significant effects of genotype (F(1,65) = 27.28, *p* < 0.0001) and age (F(4,65) = 5.32, *p* = 0.0009), but not their interaction (F < 1), with 22–65% increases in PS19 mice across the time-course. In terms of the cerebellum, we observed a significant effect of age (F(4,67) = 15.12, *p* < 0.0001), but not genotype or their interaction (both F ≤ 2.5), with higher levels at 2 and 12 months of age regardless of genotype.

#### 2.1.10. Spermidine

The levels of spermidine in five brain regions of WT and PS19 mice at five age points are presented in Figure 4B. Regarding the frontal cortex, hippocampus and parahippocampal region, we observed significant effects of genotype (FC: F(1,67) = 12.98, *p* = 0.0006; HP: F(1,66) = 39.04, *p* < 0.0001; PH: F(1,67) = 24.89, *p* < 0.0001), age (FC: F (4,67) = 7.76, *p* < 0.0001; HP: F(4,66) = 7.20, *p* < 0.0001; PH: F(4,67) = 8.77, *p* < 0.0001) and their interaction (FC: F(4,67) = 8.05, *p* < 0.0001; HP: F(4,66) = 13.53, *p* < 0.0001; PH: F(4,67) = 5.87, *p* = 0.0004), with age-related increases particularly in PS19 mice at older ages. PS19 mice displayed higher levels of spermidine in the frontal cortex at 12 months (41%), hippocampus at 8 and 12 months (33% and 75% respectively) and parahippocampal region at 8 and 12 months (both 37%) relative to their age-matched WT controls. For the striatum, there were significant effects of genotype (F(1,67) = 19.43, *p* < 0.0001) and age (F(4,67) = 7.75, *p* < 0.0001), but not their interaction (F(4,67) = 2.27, *p* = 0.07), with increased levels with age (particularly in PS19 mice at older ages) and higher levels in PS19 mice at 2 and 12 months (13% and 21% respectively) relative to their WT littermates. In terms of the cerebellum, we found no significant effects of genotype, age and their interaction (all F ≤ 2).

#### 2.1.11. Spermine

Figure 4C illustrates the levels of spermine in five brain regions of WT and PS19 mice at five age points. For the frontal cortex, striatum and cerebellum, there were significant effects of genotype (FC: F(1,66) = 6.86, *p* = 0.01; ST: F (1,63) = 5.59, *p* = 0.02; CE: F(1,66) = 5.12, *p* = 0.027), but not age or interaction (all F ≤ 2.5), with a general trend of lower levels in PS19 mice in the frontal cortex (≤9% across the time-course), striatum (10% at both 4 and 6 months) and cerebellum (15% at both 6 and 8 months). Regarding the hippocampus and parahippocampal region, we observed significant effects of genotype (HP: F(1,67) = 5.12, *p* = 0.027; PH: F(1,68) = 11.68, *p* = 0.0011) and age (HP: F(4,67) = 3.34, *p* = 0.015; PH: F(4,68) = 10.21, *p* < 0.0001), but not their interaction (both F < 2), with age-related reductions in both genotypes. Moreover, PS19 mice displayed reduced spermine levels in the hippocampus at 2–8 months (9–14%) and in the parahippocampal region at 12 months (20%) relative to their age-matched WT littermates.

#### 2.1.12. Spermidine/Spermine Ratio

The levels of spermidine/spermine ratio in five brain regions of WT and PS19 mice at five age points are presented in Figure 4D. Regarding the frontal cortex, hippocampus, parahippocampal region and striatum, there were significant effects of genotype (FC: F(1,68) = 21.51, *p* < 0.0001; HP: F(1,66) = 53.26, *p* < 0.0001; PH: F(1,67) = 101.40, *p* < 0.0001; ST: F(1,67) = 32.59, *p* < 0.0001), age (FC: F(4,68) = 8.91, *p* < 0.0001; HP: F(4,66) = 13.67, *p* < 0.0001; PH: F(4,67) = 42.61, *p* < 0.0001; ST: F(4,67) = 10.14, *p* < 0.0001) and their interaction (FC: F(4,68) = 6.39, *p* = 0.0002; HP: F(4,66) = 9.06, *p* < 0.0001; PH: F(4,67) = 25.86, *p* < 0.0001; ST: F(4,67) = 2.73, *p* = 0.036). PS19 mice displayed increased ratios in the frontal cortex at 12 months (46%), hippocampus at 8 and 12 months (63% and 66%, respectively), parahippocampal region at 8 and 12 months (43% and 92%, respectively) and striatum at 6 and 12 months (20% and 30%, respectively) relative to their age-matched WT controls. For the cerebellum, we found no significant effects of genotype, age and their interaction (all F ≤ 1).

### 2.2. mRNA Expression of the Enzymes Involved in Polyamine Synthesis and Retro-Conversion

As described above, PS19 mice (particularly at 8 and 12 months of age) displayed markedly increased levels of the polyamines putrescine and spermidine, accompanied, however, by unchanged or reduced levels of the highest order polyamine spermine. There were also genotype-related changes in the polyamine precursors L-ornithine (the product of arginase) and agmatine (the product of ADC). To understand the mechanism(s) behind these changes, we then employed the quantitative reverse transcription–polymerase chain reaction (RT-qPCR) technique to determine how mRNA expression of the enzymes involved in the de novo synthesis and retro-conversion of polyamines changed in the frontal cortex, hippocampus, parahippocampal region and cerebellum of PS19 mice at 8 and 12 months of age.

#### 2.2.1. Arginase

**Figure 5:**
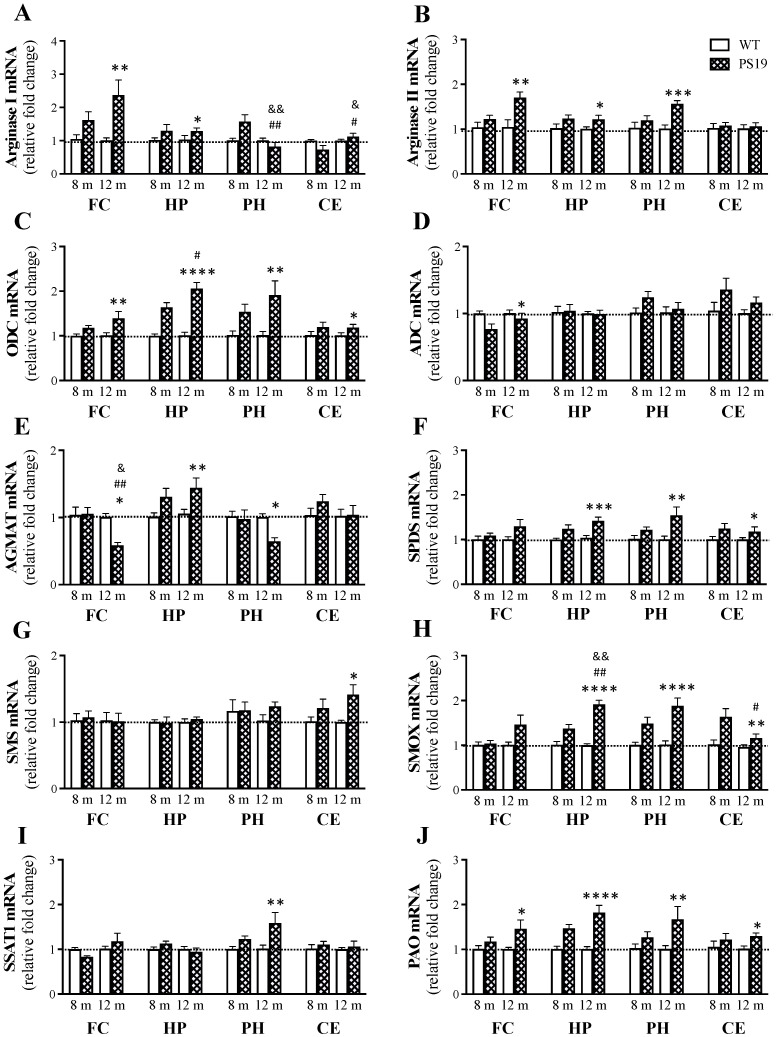
Mean (± SEM) relative fold change (2^−ΔΔCt^) of mRNA expression of arginase I (**A**), arginase II (**B**), ornithine decarboxylase (ODC, **C**), arginine decarboxylase (ADC, **D**), agmatinase (AGMAT, **E**), spermidine synthase (SPDS, **F**), spermine synthase (SMS, **G**), spermine oxidase (SMOX, **H**), spermidine/spermine-N^1^-acetyltransferase-1 (SSAT1, **I**) and polyamine oxidase (PAO, **J**) in the frontal cortex (FC), hippocampus (HP), parahippocampal region (PH) and cerebellum (CE) of wildtype (WT) and PS19 mice at 8 and 12 months (m) of age (*n* = 6–8/genotype/age). ^&^ indicates a significant genotype and age interaction at ^&^ *p* < 0.05 or ^&&^ *p* < 0.01. ^#^ indicates a significant age effect at ^#^ *p* < 0.05 or ^##^ *p* < 0.01. * indicates a significant genotype effect at * *p* < 0.05, ** *p* < 0.01, *** *p* < 0.001 or **** *p* < 0.0001.

Arginase converts L-arginine to L-ornithine, the primary precursor of the polyamine putrescine (Figure 1) [14,15,16]. Regarding arginase I (Figure 5A), in the frontal cortex, we found a significant effect of genotype (F(1,18) = 14.08, *p* = 0.0015), but not age or their interaction (both F < 2.5), with 62% and 138% increases in PS19 mice at 8 and 12 months, respectively. In the hippocampus, there was a significant effect of genotype (F(1,21) = 5.41, *p* = 0.03), but not age or their interaction (both F < 1), with a 30% increase in PS19 mice at both age points. In the parahippocampal region, we found significant effects of age (F(1,18) = 9.52, *p* = 0.0064) and its interaction with genotype (F(1,18) = 9.60, *p* = 0.0062), but not genotype (F < 2.5), with a higher level in 8-month-old PS19 mice (about 60%) relative to other groups. In the cerebellum, there were significant effects of age (F(1,21) = 5.41, *p* = 0.03) and its interaction with genotype (F(1,21) = 5.34, *p* = 0.03), but not genotype (F < 1), with a 27% reduction in 8-month-old PS19 mice relative to other groups.

For arginase II (Figure 5B), in the frontal cortex, we observed a significant effect of genotype (F(1,21) = 12.67, *p* = 0.0019), but not age (F(1,21) = 4.2, *p* = 0.053) or their interaction (F(1,21) = 3.93, *p* = 0.061), with a 70% increase in 12-month-old PS19 mice relative to their age-matched WT controls. In the hippocampus, there was a significant effect of genotype (F(1,22) = 7.14, *p* = 0.014), but not age or their interaction (both F < 1), with a 22% increase in PS19 mice at both age points. In the parahippocampal region, we found a significant effect of genotype (F(1,22) = 15.21, *p* = 0.0008), but not age (F(1,22) = 3.62, *p* = 0.07) or their interaction (F(1,22) = 4.28, *p* = 0.051), with 20% and 57% increases in 8- and 12-month-old PS19 mice respectively. In the cerebellum, there were no significant effects of genotype, age and their interaction (all F < 1).

#### 2.2.2. ODC

Figure 5C illustrates the mRNA expression levels of ODC, which converts L-ornithine to the polyamine putrescine (Figure 1) [14,15,16]. In the frontal cortex, we found a significant effect of genotype (F(1,22) = 8.86, *p* = 0.007), but not age or their interaction (both F = 1), with a 39% increase in 12-month-old PS19 mice. In the hippocampus, there were significant effects of genotype (F(1,19) = 69.19, *p* < 0.0001) and age (F(1,19) = 4.50, *p* = 0.047), but not their interaction (F(1,19) = 4.18, *p* = 0.055), with 64% and 106% increases in 8- and 12-month-old PS19 mice, respectively. In the parahippocampal region, we found a significant effect of genotype (F(1,19) = 14.88, *p* = 0.0011), but not age or their interaction (both F = 1), with 54% and 91% increases in PS19 mice at 8 and 12 months of age, respectively. In the cerebellum, there was a significant effect of genotype (F(1,20) = 4.70, *p* = 0.042), but not age or their interaction (both F < 1), with a 19% increase in PS19 mice at both age points.

#### 2.2.3. ADC

ADC converts L-arginine to agmatine (Figure 1) [14,15,16], and its mRNA levels are presented in Figure 5D. In the frontal cortex, we observed a significant effect of genotype (F(1,19) = 6.01, *p* = 0.024), but not age or their interaction (both F = 1), with a 23% reduction in 8-month-old PS19 mice. In the hippocampus, parahippocampal region and cerebellum, there were no significant effects of genotype (HP: F < 1; PH: F(1,24) = 2.89, *p* = 0.1; CE: F(1,24) = 3.58, *p* = 0.071), age (all F ≤ 1) and their interaction (all F ≤ 1).

#### 2.2.4. AGMAT

Figure 5E illustrates the mRNA levels of AGMAT, which converts agmatine to the polyamine putrescine (Figure 1) [14,15,16]. In the frontal cortex, we found significant effects of genotype (F(1,22) = 5.82, *p* = 0.025), age (F(1,22) = 8.79, *p* = 0.007) and their interaction (F(1,22) = 6.90, *p* = 0.015), with a 42% reduction in 12-month-old PS19 mice. In the hippocampus, there was a significant effect of genotype (F(1,21) = 12.54, *p* = 0.0019), but not age or their interaction (both F < 1), with 31% and 44% increases in 8- and 12-month-old PS19 mice, respectively. In the parahippocampal region, we observed a significant effect of genotype (F(1,24) = 4.31, *p* = 0.049), but not age (F(1,24) = 3.04, *p* = 0.094) or their interaction (F(1,24) = 2.63, *p* = 0.12), with a 35% reduction in 12-month-old PS19 mice. In the cerebellum, there were no significant effects of genotype, age and their interaction (all F < 1).

#### 2.2.5. SPDS

SPDS converts putrescine to the higher order polyamine spermidine (Figure 1) [17,18], and its mRNA levels are presented in Figure 5F. In the frontal cortex, we found no significant effects of genotype (F(1,21) = 3.38, *p* = 0.08), age (F = 1) and their interaction (F = 1). In the hippocampus, there was a significant effect of genotype (F(1,20) = 20.21, *p* = 0.0002), but not age (F = 2) or their interaction (F < 1), with 24% and 42% increases in PS19 mice at 8 and 12 months, respectively. In the parahippocampal region, we observed a significant effect of genotype (F(1,18) = 12.30, *p* = 0.0025), but not age or their interaction (both F = 2), with 22% and 55% increases in PS19 mice at 8 and 12 months of age, respectively. In the cerebellum, there was a significant effect of genotype (F(1,20) = 4.92, *p* = 0.038), but not age or their interaction (both F < 1), with 25% and 18% increases in 8- and 12-month-old PS19 mice, respectively.

#### 2.2.6. SMS

Figure 5G illustrates the mRNA levels of SMS, which converts spermidine to the highest order polyamine spermine (Figure 1) [17,18]. In the frontal cortex, hippocampus and parahippocampal region, we found no significant effects of genotype, age and their interaction (all F < 1). In the cerebellum, there was a significant effect of genotype (F(1,23) = 7.74, *p* = 0.011), but not age or interaction (both F < 1), with 21% and 42% increases in 8- and 12-month-old PS19 mice, respectively.

#### 2.2.7. SMOX

SMOX is involved in the retro-conversion of spermine to spermidine (Figure 1) [17,18], and its mRNA levels are presented in Figure 5H. In the frontal cortex, we found no significant effects of genotype (F(1,21) = 3.23, *p* = 0.09), age (F = 2) and their interaction (F = 2), although a 46% increase was found in 12-month-old PS19 mice relative to their age-matched WT controls. In the hippocampus, there were significant effects of genotype (F(1,19) = 67.21, *p* < 0.0001), age (F(1,19) = 11.88, *p* = 0.0027) and their interaction (F(1,19) = 12.55, *p* = 0.0022), with 37% and 92% increases in 8- and 12-month-old PS19 mice, respectively, when compared to their age-matched WT littermates. In the parahippocampal region, we observed a significant effect of genotype (F(1,19) = 29.49, *p* < 0.0001), but not age or their interaction (both F < 3), with 49% and 88% increases in 8- and 12-month-old PS19 mice, respectively. In the cerebellum, there were significant effects of genotype (F(1,22) = 13.82, *p* = 0.0012) and age (F(1,22) = 6.02, *p* = 0.023), but not their interaction (F(1,22) = 3.56, *p* = 0.072), with 64% and 16% increases in 8- and 12-month-old PS19 mice, respectively.

#### 2.2.8. SSAT1

Figure 5I illustrates the mRNA levels of SSAT1, which is a key enzyme involved in the retro-conversions of spermine and spermidine with the formation of N1-Acetylspermine and N1-Acetylspermidine, respectively (Figure 1) [17,18]. In the frontal cortex, hippocampus and cerebellum, there were no significant effects of genotype (all F < 1), age (all F ≤ 3) and interaction (all F ≤ 3). In the parahippocampal region, we found a significant effect of genotype (F(1,18) = 10.04, *p* = 0.0053), but not age or interaction (both F ≤ 2), with 23% and 59% increases in 8- and 12-month-old PS19 mice, respectively, when compared to their age-matched WT littermates.

#### 2.2.9. PAO

PAO oxidizes N1-Acetylspermine and N1-Acetylspermidine to produce spermidine and putrescine, respectively (Figure 1) [17,18], and its mRNA levels are presented in Figure 5J. In the frontal cortex, we found a significant effect of genotype (F(1,20) = 7.05, *p* = 0.015), but not age or their interaction (both F < 2), with a 46% increase in 12-month-old PS19 mice relative to their age-matched WT controls. In the hippocampus, there was a significant effect of genotype (F(1,18) = 36.33, *p* < 0.0001), but not age or their interaction (both F < 3), with 47% and 82% increases in 8- and 12-month-old PS19 mice, respectively. In the parahippocampal region, we found a significant effect of genotype (F(1,20) = 8.54, *p* = 0.0084), but not age or interaction (both F < 2), with 27% and 67% increases in PS19 mice at 8 and 12 months of age, respectively. In the cerebellum, there was a significant effect of genotype (F(1,23) = 4.84, *p* = 0.038), but not age or their interaction (both F < 1), with 22% and 30% increases in 8- and 12-month-old PS19 mice, respectively.

### 2.3. Protein Expression of the Enzymes Involved in L-Arginine Metabolism

As described above, the HPLC and LC/MS assays revealed marked genotype-related increases in the tissue concentrations of L-arginine (recycled from L-citrulline by ASS and ASL) and L-ornithine (the product of arginase). Moreover, the RT-qPCR indicated the upregulation of arginase and ODC in PS19 mice, particularly at older ages. Western blot was therefore employed to determine the time-course of changes in protein expression of the enzymes involved in the catabolism and/or biosynthesis of L-arginine and L-ornithine (arginase I, arginase II, ODC, ASS and ASL) in PS19 mice using the parahippocampal region, the brain area with more observed changes and available tissue.

**Figure 6:**
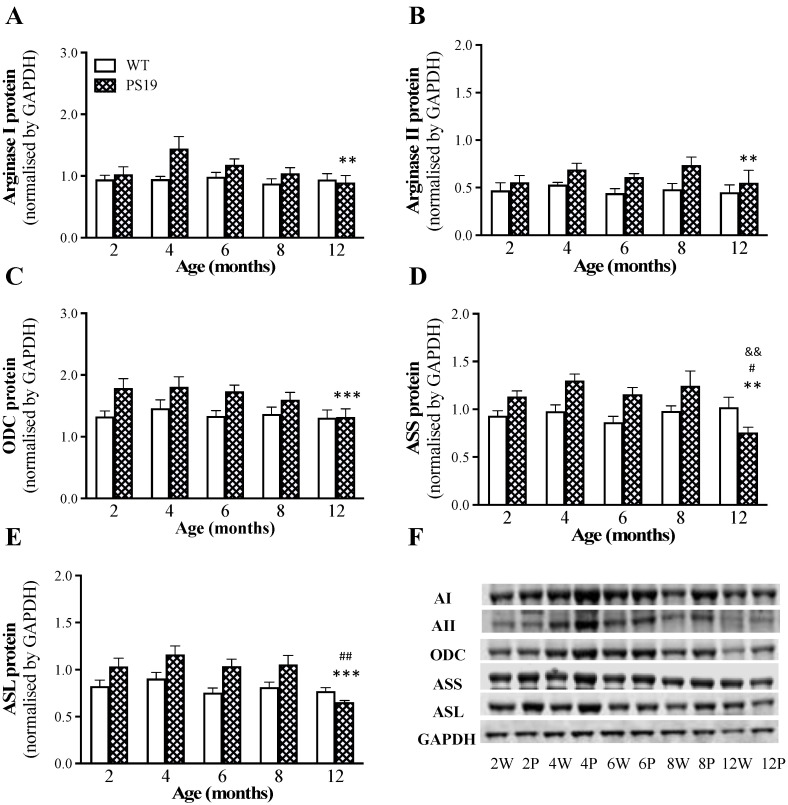
Mean (± SEM) protein expression of arginase I (AI; **A**), arginase II (AII; **B**), ornithine decarboxylase (ODC, **C**), argininosuccinate synthetase (ASS, **D**) and argininosuccinate lyase (ASL, **E**) in the parahippocampal region of wildtype (WT) and PS19 mice at 2, 4, 6, 8 and 12 months of age *(n* = 7–9/genotype/age). (**F**): representative western blots of AI, AII, ODC, ASS and ASL, as well as housekeeping protein glyceraldehyde 3-phosphate dehydrogenase (GAPDH), in wild-type (W) and PS19 (P) mice at five age points. ^&&^ indicates a significant genotype and age interaction at *p* < 0.01. ^#^ indicates a significant age effect at ^#^ *p* < 0.05 or ^##^ *p* < 0.01. * indicates a significant genotype effect at ** *p* < 0.01 or *** *p* < 0.001.

Regarding arginase I (Figure 6A), we found a significant genotype effect (F(1,64) = 7.30 *p* = 0.0088), but not age or their interaction (both F ≤ 2), with increased protein levels in 4 (53%)-, 6 (19%)- and 8 (19%)-month-old PS19 mice relative to their age-matched WT littermates. For arginase II (Figure 6B), similarly, there was a significant effect of genotype (F(1,66) = 10.96, *p* = 0.0015), but not age or their interaction (both F ≤ 1), with increased protein levels in 4 (30%)-, 6 (38%)- and 8 (53%)-month-old PS19 mice. For ODC (Figure 6C), we found a significant genotype effect (F(1,65) = 12.67, *p* = 0.0007), but no effect of age or their interaction (both F < 2), with 17–35% increases in PS19 mice at 2–8 months of age. Regarding ASS (Figure 6D), there were significant effects of genotype (F(1,63) = 9.32, *p* = 0.0033), age (F(4,63) = 2.59, *p* = 0.045) and their interaction (F(4,63) = 3.82, *p* = 0.0076), with 22–34% increases in 2–8 months-old PS19 mice but a 26% reduction in 12-month-old PS19 mice. For ASL (Figure 6E), we found significant effects of genotype (F(1,65) = 15.19, *p* = 0.0002) and age (F(4,65) = 5.03, *p* = 0.0014), but not their interaction (F(4,65) = 2.44, *p* = 0.056), with 26–37% increases in 2–8-months-old PS19 mice but a 15% reduction in 12-month-old PS19 mice.

### 2.4. Neurochemical and Behavioural Correlations

In the present study, the brain tissue samples were collected from WT and PS19 mice at 2, 4, 6, 8 and 12 months of age that underwent a battery of behavioral tests in the elevated plus-maze, open field, Y-maze and the working memory version of the Morris water maze [12]. These PS19 mice displayed hyperactivity and reduced anxiety levels with age and early and persistent spatial working memory deficits, as detailed in our previous publication [12]. We, therefore, determined neurochemical-behavioural correlations using simple linear regression, and, interestingly, several significant correlations were observed primarily in 8-month-old PS19 mice.

**Figure 7:**
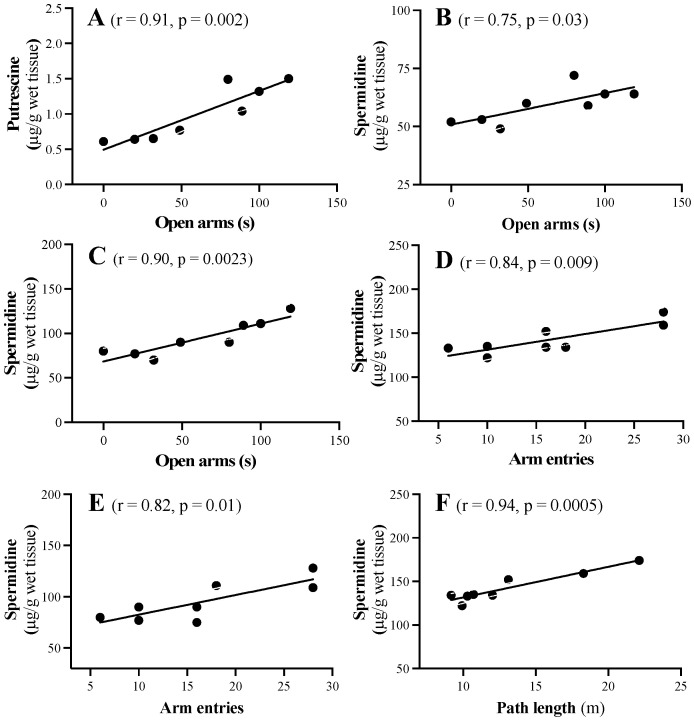
Scattergrams showing the significant correlations between the time spent in the open arms of the elevated plus-maze test and putrescine in the frontal cortex (**A**), spermidine in the frontal cortex (**B**) and parahippocampal region (**C**), between the total number of arm entries in the elevated plus-maze and spermidine in the hippocampus (**D**) and parahippocampal region (**E**) and between the total path length generated in the open field test and spermidine in the hippocampus (**F**), in 8-month-old PS19 mice.

When tested in the elevated plus-maze, PS19 mice at 8 months of age spent 85% more time in the open arms (reduced anxiety level) and made 28% more total arm entries (hyperactive) when compared to their age-matched WT controls [12]. Interestingly, the time 8-month-old PS19 mice spent in the open arms was positively correlated with their putrescine (r = 0.91, *p* = 0.002; Figure 7A) and spermidine (r = 0.75, *p* = 0.03; Figure 7B) levels in the frontal cortex and spermidine in the parahippocampal region (r = 0.90, *p* = 0.0023; Figure 7C). Moreover, their total number of arm entries was positively correlated with spermidine in the hippocampus (r = 0.84, *p* = 0.009; Figure 7D) and parahippocampal region (r = 0.82, *p* = 0.01; Figure 7E). PS19 mice at 8 months of age also generated a 20% longer path length in the open field test (hyperactive) [12]. We observed a significant positive correlation between their path length and spermidine in the hippocampus (r = 0.94, *p* = 0.0005; Figure 7F). These findings suggest that higher levels of the polyamines putrescine and/or spermidine are associated with the reduced anxiety and hyperactivity seen in 8-month-old PS19 mice [12].

## 3. Discussion

Recent human and animal research has implicated altered brain arginine metabolism in the pathogenesis of tauopathies, whereas the higher-order polyamines (spermine in particular) protect against tau fibrilization and reduce the formation of toxic tau species [25,26,28,29,30,31,38]. PS19 mice bearing the *MAPT* P301S mutation show an early and rapidly progressing neurodegenerative phenotype of tauopathy, and hence are a valuable transgenic mouse model to better understand the onset and development of tauopathies. These tau mice display soluble p-tau species in the brain along with mild spatial learning and memory deficits (that worsen with age), synaptic loss, brain deposition of tau aggregates, hippocampal neuronal loss and widespread atrophy at 2, 3, 6, 8 and 12 months of age, respectively [7,12]. Moreover, PS19 mice have altered brain arginine metabolism with a clear shift to favor the arginase-polyamine pathway, resulting in the overproduction of the polyamines putrescine and spermidine, but interestingly not spermine [28]. The present study, for the first time, carried out a time-course study across the disease progression of PS19 mice (starting at the prodromal age of 2 months) to systematically investigate how arginine metabolism (with an emphasis on the polyamine system) changed in the brains of PS19 mice at 2–12 months of age and the associations between these changes in PS19 mice and their behavioural impairments. Our results demonstrated altered arginine metabolism, the polyamine system in particular, in the PS19 brain with the upregulation of polyamine retro-conversion pathways.

### 3.1. Altered Brain Arginine Metabolism in PS19 Mice

As illustrated in Figure 1, L-arginine is a versatile amino acid with several bioactive metabolites. Therefore, we first quantified the tissue content of L-arginine and nine of its downstream metabolites in the frontal cortex, hippocampus, parahippocampal region, striatum and cerebellum of PS19 mice and their WT littermates at 2, 4, 6, 8 and 12 months of age. Regarding L-arginine, PS19 mice had significantly increased levels in the striatum (2 and 6 months), parahippocampal region (6–12 months) and hippocampus (8–12 months). In terms of its three direct metabolites L-citrulline, L-ornithine and agmatine, we found that their levels were either unchanged or increased in the brain of PS19 mice. For example, PS19 mice displayed increased levels of L-citrulline in the striatum (2 months), hippocampus (2–8 months), parahippocampal region (6 months; although reduced levels at 12 months), frontal cortex (8 months) and cerebellum (12 months). More consistent changes were seen in L-ornithine, with increased levels in PS19 mice at the age of 2 (hippocampus, parahippocampal region, striatum), 4 (frontal cortex), 6 (hippocampus and striatum), 8 and 12 months (all five regions). Regarding agmatine, PS19 mice showed increased levels in the hippocampus and parahippocampal region at 12 months of age, although a small reduction was seen in the striatum across all age points. These results clearly demonstrate genotype-related alterations in L-arginine and its three direct metabolites in PS19 mice in a region-specific and age-dependent manner, which are largely consistent with our previous study using WT and PS19 mice at 4, 8 and 12–14 months of age [28]. While the *MAPT* P301S mutation affected all three direct metabolic pathways of L-arginine, the arginase pathway appeared to be severely affected in PS19 mice as evidenced by the early and persistent increases in L-ornithine.

It is of interest to emphasize the increased L-arginine levels observed in the present study and reported in our earlier publication [28]. As the de novo synthesis of L-arginine involves the recycling of L-citrulline by ASS and ASL (Figure 1) [14,15,16], we measured their protein expression in the parahippocampal region. The findings of increased protein levels of ASS and ASL in PS19 mice at 2–8 months of age confirmed the upregulation of the L-citrulline recycling pathway, which could account for the increased L-arginine levels in PS19 mice mentioned above. Interestingly, while no significant increases in L-arginine were evident in the parahippocampal region of PS19 mice at 2 and 4 months of age, western blot showed genotype-related increases in ASS and ASL protein expression accompanied by no changes in L-citrulline. More intriguingly, PS19 mice at 12 months of age displayed increased levels of L-arginine, but reduced levels of L-citrulline (also [28]) and ASS and ASL protein expression in the parahippocampal region. It is currently unclear whether this is a compensatory mechanism aiming to normalise L-arginine.

L-ornithine can be channeled to form glutamate, which, in turn, can be metabolized to GABA and glutamine (Figure 1) [14,15,16]. In the present study, we found reduced glutamate levels in the frontal cortex (6 months), hippocampus and parahippocampal region (both 8 and 12 months) and the cerebellum (4 and 6 months) of PS19 mice. Regarding GABA, there were transient increases in the parahippocampal region (6 months), frontal cortex and striatum (both 8 months), but a decrease in the cerebellum (4 months), in PS19 mice. In terms of glutamine, PS19 mice displayed increased levels in the frontal cortex (12 months), hippocampus (6–12 months), parahippocampal region (6 months), striatum (6 and 12 months) and cerebellum (12 months). Taken together, there appeared to be a shift from glutamate to favour glutamine production in PS19 mice, particularly at older ages, which was further supported by the increased glutamine/glutamate ratios in PS19 mice at 6–12 months of age in all five brain regions examined. Similar findings were also observed in our earlier study [28], in which increased glutamine/glutamate ratios were found in 12–14-months-old PS19 mice in the five out of six brain regions examined. Collectively, these findings indicate that *MAPT* P301S mutation significantly affects the glutamate–glutamine relationship in the brain with age. It has been well documented that glutamate released by neurons is taken up by astrocytes and converted to glutamine by glutamine synthase, which can, in turn, be transported back to neurons and reconverted to glutamate by glutaminase [37,39]. Given the important role of the glutamate–glutamine cycle in neurotransmission, such widespread increases in the glutamine/glutamate ratios may contribute significantly to the behavioural deficits seen in PS19 mice [12].

### 3.2. Upregulated Arginase-Polyamine Pathway in PS19 Mice

L-ornithine can also be converted to polyamine putrescine by the rate-limiting (for polyamine biosynthesis) enzyme ODC (Figure 1) [14,17,18]. We found increased putrescine levels in the brain of PS19 mice, with sustained changes in the frontal cortex (8–12 months), hippocampus (6–12 months) and parahippocampal region (all age points) although with more transient changes in the striatum (2 and 12 months) and cerebellum (12 months). In conjunction with the genotype-related increases in L-ornithine described above, these results indicate the upregulated arginase-ODC pathway in the brain of PS19 mice, which was further supported by increased mRNA and protein expression of arginase I/II and ODC. It should be pointed out, however, that the increased arginase II and ODC mRNA expression in the parahippocampal region of 12-month-old PS19 mice were not emulated in the protein expression. Previous research has reported that protein synthesis is impaired in tauopathies, as tau disrupts translation and ribosomal function [40,41,42,43], which could explain the discrepancy between our mRNA and protein levels. Our results are also in contrast to the findings of an earlier study, in which 12-month-old rTg4510 mice harboring the *MAPT* P301L mutation displayed a reduced ODC protein level in the hippocampus [26].

Putrescine can also be synthesised by AGMAT from agmatine, decarboxylated arginine produced by ADC (Figure 1) [14,15,16]. In the present study, we found few genotype-related changes in ADC and AGMAT mRNA expression in the brain (apart from the hippocampus), indicating that the putrescine synthesis via agmatine was not largely affected in PS19 mice. However, the increased levels of agmatine and AGMAT mRNA expression in the hippocampus of PS19 mice at older ages suggest that agmatine might have contributed to the genotype-related increases in hippocampal putrescine to a certain extent.

Using post-mortem human brain tissue, earlier studies have reported that both the arginase and ODC pathways are drastically affected in AD [25,29,30,31,44]. In the present study, we found early and persistent changes in arginase I/II, L-ornithine, ODC and putrescine in the brain of PS19 mice. These findings seem to implicate an altered arginase-polyamine pathway in the pathogenesis of tauopathies. It is of interest to note, however, that arginase I upregulation induced by interleukin-4 improved learning and memory in the 3xTg mouse model of AD [45]. Sustained overexpression of arginase I also markedly reduced tau pathology and kinases involved in tau phosphorylation and mitigated hippocampal atrophy in rTg4510 tau mice [38], whereas ODC upregulation appears to be detrimental in PS19 mice [25]. ODC antizyme is a small regulatory protein that inhibits ODC activity, promotes ODC degradation and down-regulates polyamine uptake, and itself can be regulated by agmatine and antizyme inhibitors [46,47,48]. It has been shown that these ODC regulators (ODC antizymes and antizyme inhibitors) are upregulated in AD brains [25]. Interestingly, overexpression of antizyme inhibitor 2 (hence increased ODC activity) augmented tau neuropathology and cognitive impairments in PS19 mice [25]. In view of these results, further work is required to better understand the implication of ODC dysregulation in the pathogenesis of tauopathies.

### 3.3. Polyamine System Dysfunction in PS19 Mice

The higher-order polyamines spermidine and spermine can be formed through de novo synthesis by SPDS (from putrescine) and SMS (from spermidine), respectively, or channelled back through the retro-conversion pathways via SMOX and SSAT1/PAO (Figure 1) [17,18]. Alike to putrescine, spermidine levels were drastically increased in the frontal cortex (12 months), hippocampus and parahippocampal region (both 8 and 12 months), and striatum (2 and 12 months) of PS19 mice. Since the increased spermidine in PS19 mice was found alongside increased SPDS mRNA expression in the hippocampus and parahippocampal region, putrescine is likely an important source for increased spermidine levels in these regions. Regarding spermine, intriguingly, we found no changes or mild reductions in PS19 mice in all five brain regions examined and no parallel genotype-related increases between spermidine and spermine. When SMS was investigated, its mRNA expression levels were unaltered in the brain of PS19 mice, except for the cerebellum. The spermidine/spermine ratio is normally tightly conserved for normal cellular function [34,35,36]. However, the differential effects of the *MAPT* P301S mutation on the two higher-order polyamines resulted in the increased spermidine/spermine ratios in PS19 mice primarily in the cerebral brain areas in an age-dependent manner.

The increased putrescine and spermidine levels, but decreased or unchanged spermine concentrations, alongside unchanged SMS in the cerebral brain areas of PS19 mice, led us to investigate how the enzymes involved in the direct (SMOX) and indirect (SSAT1 and PAO) polyamine retro-conversion pathways changed in the brain. Our RT-qPCR work revealed genotype-related increases in the mRNA expression of SMOX (in the hippocampus, parahippocampal region and cerebellum), SSAT1 (the parahippocampal region only) and PAO (in all four regions examined), therefore confirming increased polyamine retro-conversion in PS19 mice. Using the whole brain tissue, moreover, Sandusky-Beltran et al. [26] reported increased levels of putrescine, SPDS, SMOX and SSAT1 expression (although reduced SMS expression) in 8–12-month-old rTg4510 mice. Taken together, these findings demonstrate that *MAPT* mutations lead to polyamine system dysfunction in the brain, which involves the dysregulation of the de novo synthesis pathway and the upregulation of both the direct and indirect retro-conversion pathways. It should be noted that cellular polyamine transporters constitute another key regulator of the polyamine system and have been genetically linked to the early onset forms of Parkinson’s disease [49]. Future research is required to explore their role in the dysregulation of the polyamine system in tauopathies.

Polyamines are normally tightly regulated through complex feedback mechanisms to maintain and modulate their key physiological functions, including DNA, RNA and protein synthesis, cell proliferation and differentiation, microtubule assembly and stabilization, as well as neurotransmitter receptor regulation [17,18,20,21,22,23,24]. However, transient increases in polyamines (known as the polyamine stress response, or PSR) can occur following exposure to stress signals to promote cell survival mechanisms [50,51,52]. The results of the present study demonstrate the presence of a sustained PSR in PS19 mice, as evidenced by the prolonged elevation of putrescine and spermidine and increased spermidine/spermine ratio in the brain. It has been shown that polyamines can regulate microtubule homeostasis and prevent tau fibrillization [22,23,24,25,26]. However, high levels of spermidine can induce SSAT1 to initiate the polyamine retro-conversion via the indirect pathway [53,54], and acetylated polyamines can exacerbate tau aggregation and seeding [25,26], hence augmenting tau pathology. Moreover, the polyamine retro-conversion enzymes produce toxic by-products, including highly reactive oxygen species, aldehydes and acrolein [32,33,55,56,57,58], in addition to acetylated polyamines, which are elevated in tauopathies and are associated with the accumulation of pathological tau species [25,26]. Reduced spermine level as a consequence of the polyamine retro-conversion could also be devastating for the brain, as it is the most potent polyamine regulating cell survival, neurotransmission, microtubule polymerisation and stabilisation and combating tau pathology by reducing tau aggregation into its more toxic fibrillar and oligomeric forms [21,22,24,25,26,59]. Furthermore, high levels of polyamines can be neurotoxic, due to their modulatory roles on N-methyl-D-aspartate (NMDA) receptors [60,61,62,63]. To this end, the sustained PSR in PS19 mice is likely detrimental. Taken together, our study supports the idea that tauopathies are a result of a chronic dysregulated PSR [26,51], although it needs to be further validated and the underlying mechanisms remain to be investigated.

It has been well documented that polyamines are critically involved in learning and memory processes [17,18,19]. PS19 mice used in the present study displayed hyperactivity and reduced anxiety levels with age, and early and persistent spatial working memory deficits [12]. While there were no direct correlations between the polyamines and working memory deficits, higher levels of the polyamines putrescine and/or spermidine appeared to be associated with reduced anxiety and hyperactivity seen in 8-month-old PS19 mice. The polyamine system has been implicated in various mental disorders, with genetic polymorphisms in SSAT1 and SMS linked to anxiety disorders and altered polyamine levels associated with psychological stress [64,65,66,67]. It is of interest to note that hyperactivity is a major phenotype of SMS-deficient mice, which exhibit reduced spermine levels and an increased spermidine/spermine ratio in the brain [36,68].

### 3.4. Conclusions

The present study, for the first time, systematically investigated the time-course of changes in brain arginine metabolism, particularly the polyamine system, in PS19 mice at 2–12 months of age (from prodromal to severe disease-like stages). Consistent with our earlier research [28], the *MAPT* P301S mutation affected brain arginine metabolism drastically in a region-specific and age-dependent manner. Importantly, our findings largely replicated genotype-related neurochemical changes and a shift of arginine metabolism towards the arginase-polyamine pathway [28], further demonstrating the upregulation of the L-citrulline recycling, arginase-ODC and polyamine retro-conversion pathways (Figure 1), the association between altered polyamines (putrescine and spermidine) and the reduced anxiety and hyperactivity behaviours observed in PS19 mice at 8 months. The brain regions affected most severely in PS19 mice appeared to be the hippocampus and the parahippocampal region, which are important for cognition and mood and are affected early and severely in several tauopathies [69,70,71,72,73,74]. While most genotype-related changes observed in this study often occurred at the older age points, PS19 mice at 2 months of age displayed increased levels of L-ornithine in three brain regions, alongside putrescine, spermidine and the enzymes ODC, ASS and ASL. It is of interest to note that PS19 mice at such young ages already showed mild working memory deficits and the accumulation of soluble p-tau species in the brain in the absence of overt neuronal or synaptic loss [7,12]. Hence, our findings further implicate altered arginine metabolism, particularly the polyamine system (including a sustained PSR), in the pathogenesis of tauopathies. We propose that initially the PSR was induced in the PS19 mice perhaps as a neuroprotective response to counter the accumulation of p-tau by stabilising microtubules and/or sequestering tau in its less toxic unaggregated forms. With age, the polyamine retro-conversion pathways were upregulated in the PS19 mice, leading to the prolonged overproduction of putrescine and spermidine. The sustained PSR and the toxic polyamine retro-conversion by-products augmented tau pathology and cognitive dysfunction. Future research is required to better understand the functional significance of PSR in tauopathies and to explore the preventive and/or therapeutic opportunities by targeting the polyamine system.

## 4. Materials and Methods

### 4.1. Animals

Male P301S *MAPT* transgenic (PS19) mice (B6;C3-Tg(Prnp-MAPT*P301S)PS19Vle/J; stock number: 008169; Jackson Laboratory) and C57BL/6J female mice were crossed to produce PS19 and WT offspring that were confirmed by tail tip genotyping. The present study used male PS19 mice and their age-matched WT littermates at 2, 4, 6, 8 and 12 months of age (*n* = 7–16/genotype/age), which underwent a battery of behavioural tests and cerebral blood flow assessments in our previous study [12]. All animals were group-housed in 15 × 20 × 38 cm^3^ polypropylene individually ventilated cages, maintained on a 12 h light/dark cycle regime (light on at 7:00), and provided *ad libitum* access to food and water. Animals’ body weights and general health conditions were closely monitored. All experimental procedures were carried out in accordance with the regulations of the University of Otago Animal Ethics Committee. Every attempt was made to reduce the number of animals used and to minimise their suffering.

### 4.2. Brain Tissue Collection

Brain tissue collection was performed 2–3 days after the completion of the last behavioural test following a cerebral blood flow assessment [12,75,76,77]. Each animal was transcardially perfused with ice-cold saline, and the brain was rapidly removed and kept in cold saline (4 °C) for at least 45 s. The frontal cortex (FC), whole hippocampus (HP), parahippocampal region (PH containing the entorhinal, perirhinal and postrhinal cortices), striatum (ST) and cerebellum (CE) were dissected freshly on ice from each hemisphere, immediately snap-frozen on dry ice and stored at −80 °C for the HPLC and LC/MS assays, RT-qPCR and western blot.

### 4.3. HPLC and LC/MS Assays

Brain tissue samples (from the FC, HP, PH, ST and CE) were weighed, homogenised in ice-cold 10% perchloric acid (∼50 mg wet weight per ml) and centrifuged at 10,000× *g* for 10 min. The perchloric acid extracts (supernatants) were then stored at −80 °C until the HPLC and LC/MS assays. The brain tissue concentrations of L-arginine, L-citrulline, L-ornithine, glutamate, glutamine, GABA, spermidine and spermine were measured using HPLC, while agmatine and putrescine concentrations were measured by a highly sensitive LC/MS method as described in detail in our previous publications [28,31,76,78]. High-purity external and internal standards were used (Sigma, Sydney, Australia) and all other chemicals were of analytical grade. For each brain region, the samples from the PS19 and WT mice at all five age points were assayed in duplicate simultaneously in a counterbalanced manner. The concentrations of L-arginine and its nine downstream metabolites in each tissue were calculated with reference to the peak area of external standards, and values were expressed as μg/g wet tissue. The experimenters were blind to the grouping information at the time of the assay.

### 4.4. RNA Extraction, cDNA Synthesis and RT-qPCR

RT-qPCR was employed to measure the mRNA expression of ADC, AGMAT, arginase I and II, ODC, SPDS, SMS, SMOX, SSAT1 and PAO in the FC, HP, PH and CE tissue of 8- and 12-month-old PS19 mice and their age-matched controls. Total RNA from brain tissue (20–30 mg) was extracted using a mirVana™ PARIS™ Protein and RNA Isolation System (Thermo Fisher Scientific, AM1556) according to the manufacturer’s instructions. RNA quality and quantity were determined by spectrophotometric analysis (NanoDrop™ ND-1000, Thermo Fisher Scientific, Waltham, MA, USA). A High Capacity RNA-to-cDNA kit (Applied Biosystems, 4387406) was used to convert the RNA to cDNA as per the vendor’s instructions on a Biometra TAdvanced PCR Thermal Cycler (Analytik, Jena, Germany). RT-qPCR (triplets per sample) was performed with cDNA as a template using a Light Cycler^®^ 480 SYBR^®^ Green I Master kit (Roche, 04707516001, Basel, Switzerland) on a ViiA™ Real-Time PCR System (Applied Biosystems, Waltham, MA, USA) in a final volume of 10 μL. The cycling conditions were 95 °C for 10 min, 40 cycles of 30 s each at 95 °C, 60 °C and 72 °C, followed by a melt curve. The primers used are depicted in Supplementary Table S1. Efficiency of the primers was tested using the standard curves made from the same batch of cDNA. Analysis of the melt curve indicated no non-specific amplification or primer dimer formation. Plate-to-plate variations were corrected to a calibrator sample, prepared from an independent cDNA sample, not part of the cohort. Relative mRNA levels were normalized against the house-keeping genes; glyceraldehyde 3-phosphate dehydrogenase (GAPDH) and hypoxanthine-guanine phosphoribosyltransferase (HPRT), and presented as 2^−ΔΔCt^.

### 4.5. Western Blot

Brain samples were homogenised in protease-inhibitory buffer (50 mM Tris-HCl (pH 7.4), 10 μM phenylmethylsulfonyl fluoride, 15 μM pepstatin A and 2 μM leupeptin) on ice, and centrifuged at 12,000× *g* for 10 min at 4 °C. The supernatant was collected and stored at −80 °C and the protein concentration was determined using the Bradford method [79]. The protein expression of arginase I and II, ASL, ASS and ODC in the parahippocampal region of PS19 mice in relation to their age-matched controls was then determined using western blots, as previously described [77]. All the samples were mixed with gel-loading buffer, containing 50 mM Tris-HCl, XT sample buffer (Bio-Rad Laboratories, 610791, Hercules, CA, USA) and reducing agent (Bio-Rad, 1610792), with the final protein concentration equalized to 2 mg/mL and boiled for 5 min. The samples (5–10 μL) were loaded into a Criterion^TM^ XT 4–12% gradient Bis-Tris gel (Bio-Rad, 3450125), along with pre-stained protein markers (10–250 kDa; Bio-Rad) and a biological control sample. The loading order was counter-balanced between the samples from the genotype/age groups for each brain region. The separated proteins were transferred onto nitrocellulose blotting membranes using a transblotting apparatus (Bio-Rad) and then blocked with 5% bovine serum albumin (BSA) in Tris-buffered saline with 0.1% Tween-30. The membranes were then incubated with primary polyclonal goat antibody against arginase I (1:1000; Santa Cruz, sc-18355), ASL (1:1000; Santa Cruz, sc-68250) or ASS (1:1000; Santa Cruz, sc-46066), or monoclonal mouse antibody against arginase II (1:1000; Santa Cruz, sc-271443), or ODC (1:1000; Abcam, ab66067) overnight at 4 °C. As a loading control, the monoclonal mouse or rabbit antibody against the housekeeping protein GAPDH (1:100,000; Abcam, ab8245 or ab181602, respectively) was used. The following day, the membranes were probed with IRDye^®^ 680RD goat anti-mouse IgG antibody, IRDye^®^ 680RD goat anti-rabbit IgG antibody, IRDye^®^ 680RD donkey anti-mouse IgG antibody, IRDye^®^ 800 CW goat anti-mouse IgG antibody or IRDye^®^ 800 CW donkey anti-goat IgG antibody (all 1:10,000; LI-COR Biosciences, Lincoln, NE, USA). Detection of the immunoreactive signal bands was performed using an Odyssey^®^ CLx Imager (LI-COR). Signals were quantified with Odyssey CLx Image Studio software (LI-COR), normalized with the corresponding GAPDH loading controls and biological controls to account for inter-gel variation. The experimenters were blind to the grouping information at the time of the assay and analysis.

### 4.6. Statistical Analysis

All data from Section 2.1, Section 2.2 and Section 2.3 were analysed using two-way analysis of variance (ANOVA) to determine the effects of genotype, age and their interaction, followed by post-hoc tests (Fisher’s LSD for Section 2.1 and Section 2.3 or Ŝídák tests for Section 2.2). Simple linear regression was used to determine the correlations between the neurochemical variables and previously reported behavioural data [12] in Section 2.4. Statistical analyses were performed using GraphPad Prism software (Version 9.3.1), and all data were presented as mean and standard error of the mean (mean ± SEM). The level of significance was set at *p* < 0.05 for all comparisons.

## Data Availability

The data presented in this study are available in the article.

## Supplementary Materials

The following supporting information can be downloaded at: https://www.mdpi.com/article/10.3390/ijms23116039/s1.
