# Supplementary material for: Altered Brain Arginine Metabolism and Polyamine System in a P301S Tauopathy Mouse Model: A Time-Course Study
Source: Int J Mol Sci. 2022 May 27;23(11):6039. doi: 10.3390/ijms23116039 (PMC9181759; doi:10.3390/ijms23116039)
Supplement: Supplementary file 1 [file ijms-23-06039-s001.zip › ijms-1724516-supplementary.pdf]

Supplementary Materials

Scheme S1. Primers used for quantitative reverse transcription-polymerase chain reaction (RT-qPCR).

Gene	Species	Forward (5′-3′)	Reverse (5′-3′)
Arginase-I	Mouse	CTC CAA GCC AAA GTC CTT AGA G	AGG AGC TGT CAT TAG GGA CAT C
Arginase-II	Mouse	CTC CCT GCC AAT CAT GTT CCT	CCT CTG CCT TTT GCC AAT CA
Ornithine decarboxylase	Mouse	ACA TGG CAA CAG AAG TTG G	GAG CTG GGT TGA TTA CAC TG
Polyamine oxidase	Mouse	CAA GGA CTT ACC GAC CGC AT	TGG GTT TCC TGT CAC TCT CC
Spermine oxidase	Mouse	TGG CCT GTA GTC GTG GAG T	GCA TGG CCT AAA GAA ACT GGT G
Spermine synthase	Mouse	TCA GTT CCT GCT AAG CAC C	ACC TCT TTC TTC CTC CTT TCC
Spermidine synthase	Mouse	GTC CAG TGC GAG ATT GAT G	TTG AGC TGG AGA AGC CAA C
Spermidine/spermine-N(1)-acetyltransferase-1	Mouse	GGC GGG CTT TCA TCC TTA C	CCC AAC AAT GCT ATG TCC TTC AG
Arginine decarboxylase	Mouse	TGC CTT CAC TGT GGC TGT CA	AGA CCA GCC AGT CCC CTA CT
Agmatinase	Mouse	TGC ACA GCA AGC GAG TGG TAC A	GGA CCA GTG ACT TCA TCC AAC AG
Glyceraldehyde 3-phosphate dehydrogenase	Mouse	CGT CCC GTA GAC AAA ATG GT	TTG ATG GCA ACA ATC TCC AC
Hypoxanthine-guanine phosphoribosyltransferase	Mouse	CTT CCT CCT CAG ACC GCT TTT	CTG GTT CAT CAT CGC TAA TCA CG
